# Posterior Reversible Encephalopathy Syndrome in the Postpartum Period: A Rare Entity With Atypical Presentation

**DOI:** 10.7759/cureus.82728

**Published:** 2025-04-21

**Authors:** Auriane Leslie Kouam Djembi, Marie-Anne Labaisse, Thibault Warlop, Valery Bogne Kamdem

**Affiliations:** 1 Emergency Medicine, Centre Hospitalier de Wallonie Picarde, Tournai, BEL; 2 Radiology, Centre Hospitalier de Wallonie Picarde, Tournai, BEL; 3 Neurology, Centre Hospitalier de Wallonie Picarde, Tournai, BEL; 4 Obstetrics and Gynecology, Hôpital Riviera-Chablais, Vaud-Valais, CHE

**Keywords:** brain capillary leak syndrome, diffusion-weighted image (dwi), neurologic disorder, posterior reversible encephalopathy syndrome (pres), postpartum preeclampsia, preeclampsia-eclampsia

## Abstract

Posterior reversible encephalopathy syndrome (PRES) is a rare condition characterized by clinical and brain imaging criteria. It is most often associated with pregnancy in the postpartum period and has a higher incidence in primiparous patients. Its presentation includes headaches, altered consciousness, seizures, visual disturbances, and specific radiological signs, particularly vasogenic edema of the subcortical white matter, primarily located in the posterior cerebral hemispheres. While PRES in obstetrics is most commonly associated with preeclampsia or, more frequently, eclampsia, we report a case occurring in a normotensive patient. Our case involves a primiparous patient in the postpartum period who presented with a wide range of severe neurological symptoms, including transient cortical blindness and severe memory loss correlated to lesions in atypical regions, such as the temporal lobes and bilateral hippocampi and thalami. The distribution of lesions, the apparent diffusion coefficient (ADC) and diffusion-weighted imaging (DWI) MRI mapping allowed a differentiation of PRES from early cerebral ischemia, thus playing an essential role in management. This case highlights the need to consider PRES in postpartum patients with significant neurological symptoms, regardless of blood pressure or proteinuria status. Early recognition and diagnosis are essential for optimal outcomes, given the reversible nature of the syndrome with timely intervention.

## Introduction

Posterior reversible encephalopathy syndrome (PRES) was first described in 1996 by Hinchey et al. [1]. It is characterized by (sub)acute neurological symptoms such as headaches, altered consciousness, visual disturbances (ranging from blurred vision to hemianopia or even cortical blindness), seizures, and vasogenic edema mainly localized in the parieto-occipital lobes [1,2]. 

This syndrome has been described in association with several pathologies, the most common being hypertensive encephalopathy, eclampsia and immunosuppressive and cytotoxic therapies, which is the most frequent etiology. Although its prevalence is unknown, PRES affects individuals of all ages, mostly women. Its incidence in eclampsia reaches 22.5% in some series [3,4]. Hypertension is reported in the majority of cases, although blood pressure may be normal or only slightly elevated in 20 to 30% of cases [5]. The physiopathology in normotensive patients is still poorly understood.

However, this clinical presentation is not specific to PRES and can be observed in other medical conditions, such as reversible cerebral vasoconstriction syndrome (RCVS), hypertensive encephalopathy, cerebral venous thrombosis, cerebrovascular syndrome, particularly the top-of-the-basilar syndrome with bilateral posterior infarction, acute demyelinating encephalomyelitis (commonly postinfectious or postvaccinial), toxic or metabolic encephalopathy, infectious encephalitis, paraneoplastic or autoimmune encephalitis, and vasculitis [2]. Therefore, neuroimaging, especially diffusion-weighted imaging (DWI), apparent diffusion coefficient (ADC) and fluid-attenuated inversion-recovery (FLAIR) MRI mapping, is essential for the diagnosis and management of PRES. Appropriate management is associated with limited neurological morbidity and, in most cases, complete clinical recovery.

This case illustrates an atypical presentation of PRES in a normotensive patient during the puerperal period, marked by extensive brain lesions, including the unusual involvement of both temporal lobes, as well as bilateral hippocampal and left thalamic involvement associated with severe memory impairment.

## Case presentation

A 34-year-old primiparous woman, at 38 weeks and two days pregnant, with no previous medical history and treated with Ursochol for gestational cholestasis, was admitted to the gynecology department for labor induction due to acute thrombocytopenia and persistent liver test disturbances (Table 1). Her vital signs were normal (Blood Pressure: 121/85 mmHg, Heart Rate: 86 bpm, Temperature: 36.6 °C), and no proteinuria was detected.

**Table 1:** Patient's blood test on admission.

Biology	Patient on Admission	Reference Range
Hemoglobin	13.1	11.7 - 15.50 g/dl
Platelets	127000	150000 - 400000 /mm^3^
Leukocytes	7700	4500 - 11000 / mm^3^
C-reactive protein (CRP)	< 0.6	< 5 mg/L
PT (Prothrombin Time)	>100	> 70%
APTT (Activated Partial Thromboplastin Time)	25	25.1 - 37 seconds
Fibrinogen	479	200 - 393 mg/dL
INR (International Normalized Ratio)	0.9	< 1.5
Urea	27	15 - 40 mg/dL
Uric acid	5.9	2.4 - 5.7 mg/dL
Creatinine	0.8	< 0.9 mg/dL
Total Bilirubin	1	< 1.1 mg/dL
Direct Bilirubin	0.5	< 0.3 mg/dL
Aspartate aminotransférase (ASAT)	29	< 32 U/L
Alanine aminotransférase (ALAT)	19	< 33 U/L
γ-Glutamyl transpeptidase	60	< 36 U/L
Bile Acid	29	< 10 µmol/L

This clinical picture is not consistent with typical preeclampsia. Fetal well-being was preserved. Labor induction was performed with misoprostol, and she received an epidural anesthesia consisting of ropivacaine and sufentanil.

A few hours in labor, an emergency cesarean section under general anesthesia was performed due to pathological fetal monitoring (decelerations), and was complicated by severe postpartum hemorrhage with total blood loss estimated at 3.7 liters, requiring admission to the intensive care unit. The patient received hemodynamic support with crystalloids, vasopressors (a continuous intravenous infusion of norepinephrine titrated to maintain a mean arterial pressure of at least 65 mmHg), and transfusion guided by rotational thromboelastometry (six units of red blood cells, four units of fresh frozen plasma, one unit of platelets, and two grams of fibrinogen), achieving hemodynamic stability within 24 hours. She was weaned off the ventilator, and vasopressors were discontinued.

Forty-eight hours after the delivery, the patient experienced a brief loss of consciousness (two to three minutes) associated with generalized tonic movements and ocular revulsion without any clonic movements. Her vital signs remained normal. Upon awakening, she was amnesic of the late events (visit of her husband and baby 30 minutes before) and complained of holo-cranial headache, nausea, and asthenia. Neurological examination showed no signs of meningeal irritation or sensory-motor lateralization. Her vision was normal, pupils were isochoric and reactive, and the cranial nerve examination was normal (in particular, no facial paralysis or oculomotor disorder). Muscle tone was diminished but symmetrical in both the upper and lower limbs. The plantar cutaneous reflexes were normal, and the deep tendon reflexes were brisk but symmetrical. Her vitals were still normal.

We performed a cerebral CT scan with contrast, which revealed no abnormalities. The EEG displayed a slowed trace without asymmetry, focal points, or epileptiform abnormalities. The patient was empirically given intravenous levetiracetam (bolus of 40 milligrams/kilogram followed by 2 grams/24 hours) and magnesium sulfate (12 grams/24 hours).

Fifty hours after the delivery, she reported bilateral blindness, and her memory loss worsened (spatio-temporal disorientation, inability to recall her delivery and lack of knowledge about her profession).

**Figure 1:**
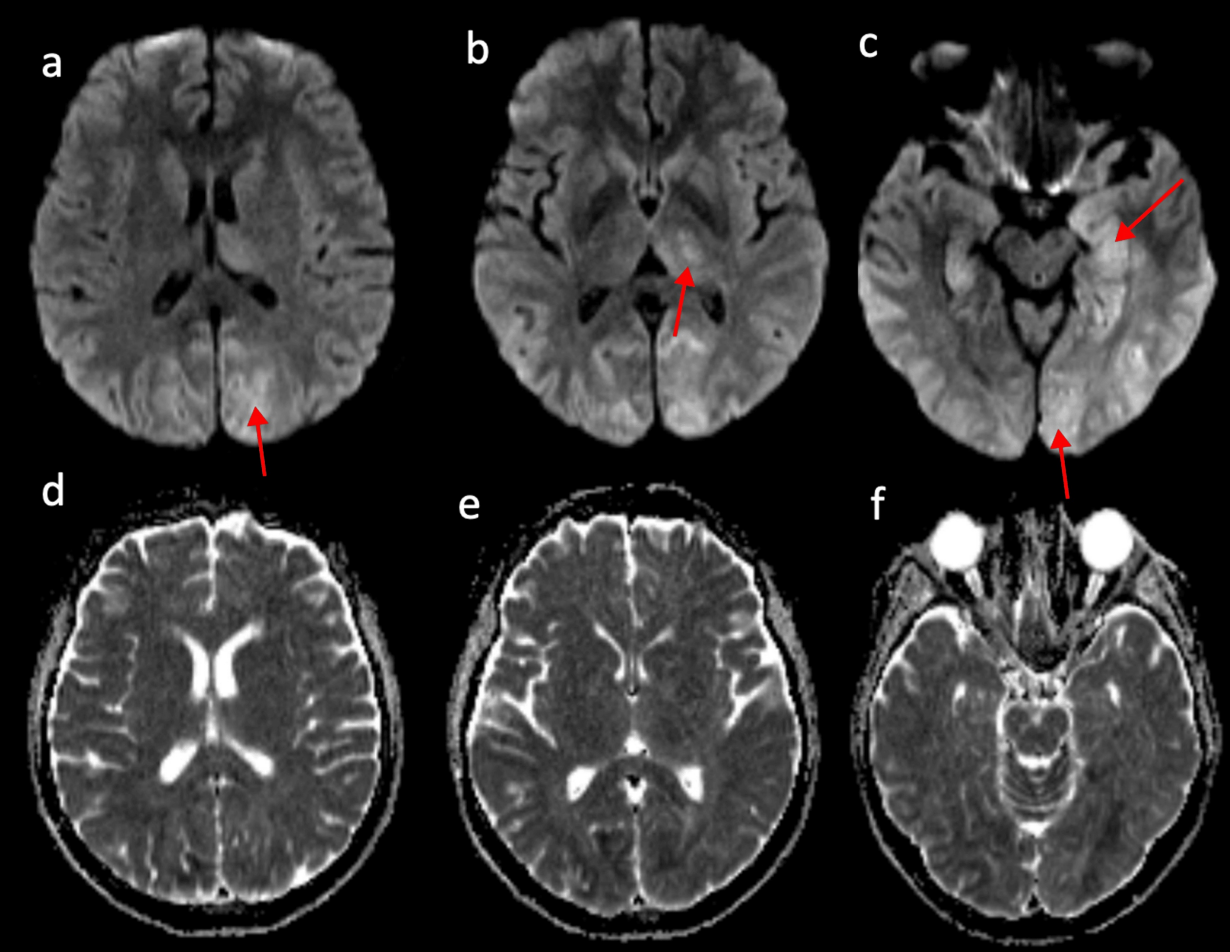
Axial DWI MR images (a,b,c) and ADC map (d,e,f) at the onset. These images reveal acute ischemic lesions in the posterior regions of both cerebral hemispheres, indicative of Posterior Reversible Encephalopathy Syndrome. The DWI images display high signal in the cortex and subcortical white matter of the occipital, parietal, and temporal lobes. Additionally, high signals are noted in the left thalamus and the right part of the splenium of the corpus callosum. The low signal areas on the ADC images reflect cytotoxic edema within vasogenic edema. DWI: Diffusion-Weighted Imaging; ADC: Apparent Diffusion Coefficient

**Figure 2:**
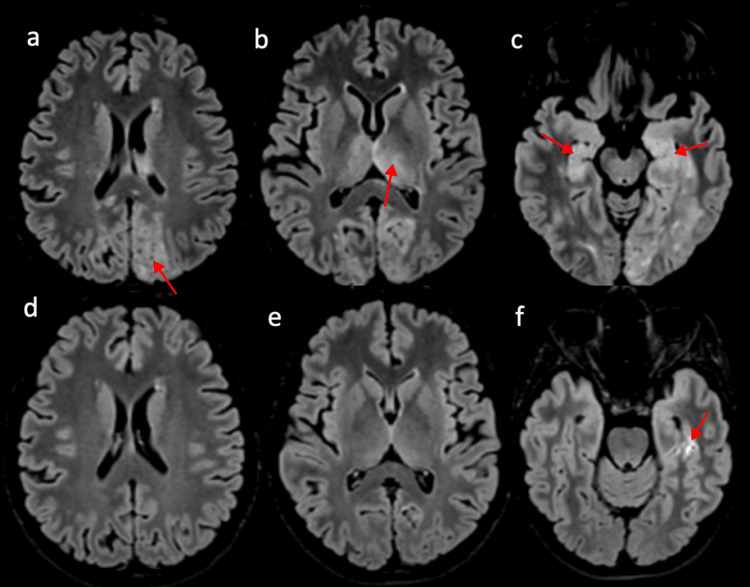
Axial flair images at the onset ( a,b,c) and two months later (d,e,f). The initial FLAIR images show hyperintense cortico-subcortical edema in the parietal, occipital and temporal lobes, with the most severe involvement affecting the left temporal lobe. There is also central involvement with thalamic and posterior callosal hypersignal. The memory impairment is likely correlated with the involvement of both hippocampi (image c, solid arrows). FLAIR images taken two months later showed resolution of all hyperintensities, except in the left inferior temporal cortex, indicating chronic ischemia (image f). FLAIR: Fluid-Attenuated Inversion-Recovery

A cerebral MRI revealed recent ischemic lesions characterized by hyperintensity on FLAIR and diffusion-weighted sequences. These lesions display areas of restricted diffusion (decreased ADC) occupying the temporal and occipital lobes, predominantly on the left, the splenium of the corpus callosum, the left pre- and postcentral gyrus, the left thalamus, the left capsulo-lenticular junction, as well as the protuberance bilaterally and the cerebellum predominantly on the left (Figure 1, Figure 2A-2C).

The involvement of both hippocampi (Figure 2C, solid arrows) and the left thalamus (Figure 2B) explains the severity of memory loss.

**Figure 3:**
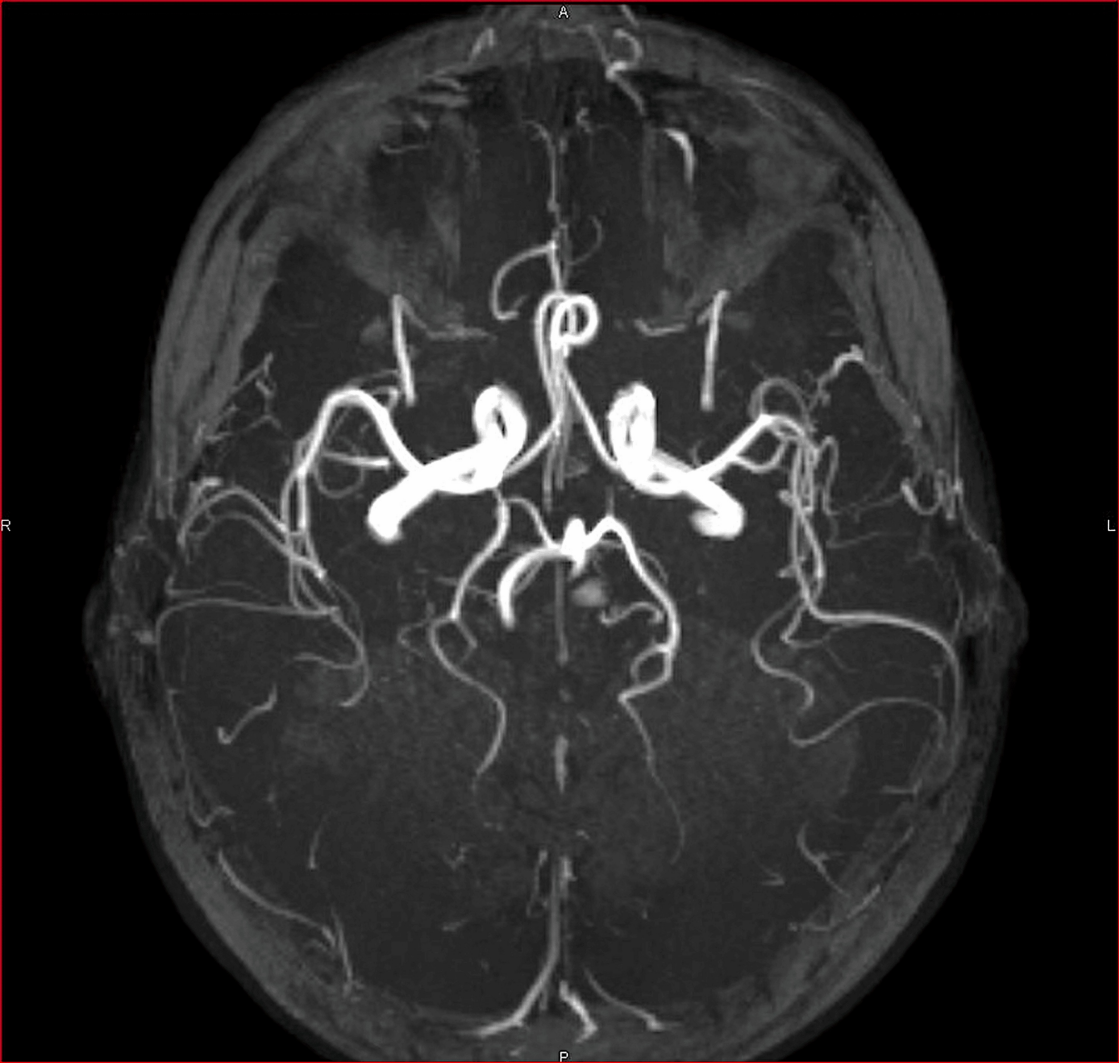
Axial Maximum Intensity Projection (MIP) of the 3D time of flight MR angiogram. Cerebral angiography shows no stenosis or vascular occlusion of the arteries of the circle of Willis.

Magnetic resonance angiography showed normal caliber of the arteries of the Willis polygon (Figure 3). The findings are consistent with a diagnosis of PRES.

The absence of arterial vasoconstriction rules out a reversible cerebral vasoconstriction syndrome. Additionally, no venous thrombosis was detected on MRI.

Outcome

The patient gradually regained her vision within five days of losing consciousness, although her memory loss persisted. Osteotendinous reflexes normalised within a week.

A follow-up EEG on Day 10 revealed an epileptiform abnormality in the left frontotemporal region, justifying the continuation of antiepileptic treatment. 

Three weeks later, persistent retrograde amnesia (related to her delivery and her stay in the intensive care unit) and short-term memory impairment were observed.

Two months later, a follow-up brain MRI showed a chronic ischemic lesion in the left inferior temporal gyrus and the complete resolution of the other abnormalities previously described (Figure 2D-2F). Mild short-term and long-term memory loss persisted without affecting the quality of life. The EEG showed a slightly irritative rhythm in the right temporal region, which led to the continuation of antiepileptic treatment.

## Discussion

Posterior reversible encephalopathy syndrome is characterised by a rapid onset of symptoms, which can evolve within a few hours to days. It is also referred as brain capillary leak syndrome or hyperperfusion encephalopathy due to its heterogeneous nature. The pathogenesis is incompletely understood, but it is believed to result from endothelial dysfunction and impaired cerebral autoregulation [1,2].

Endothelial dysfunction has been linked to PRES associated with preeclampsia or cytotoxic therapies [1,3].

In preeclampsia, the secretion of cytotoxic trophoblastic factors may provide the initial stimulus. Biomarkers indicating endothelial damage such as lactate dehydrogenase, abnormal red blood cells and more specific ones including fibronectin, tissue plasminogen activator, thrombomodulin, endothelin-1, and, in particular, von Willebrand factor can be detected before symptoms emerge. The severity of cerebral edema in PRES seems to correlate more strongly with these markers than with fluctuations in blood pressure [2]. The syndrome frequently manifests during the postpartum period (55% of cases) rather than during pregnancy and has a higher incidence in primiparous patients (81% of cases) [1,6]. Some studies suggest that PRES might serve as a neurological indicator of eclampsia, even in the absence of traditional signs like hypertension or proteinuria. Additionally, blood pressure levels in preeclamptic or eclamptic patients with PRES are generally lower than those observed in patients who develop the syndrome due to other causes [2,7]. 

Few cases of PRES occurring in pregnant women without hypertension, preeclampsia or eclampsia, as observed in our patient, have been reported. However, the pathophysiology remains poorly understood. Proposed mechanisms include immune system alterations, placental abnormalities, hormonal fluctuations, oxidative stress, dietary factors, and genetic susceptibility, among others [8]. Further studies are needed to elucidate the pathophysiology of pregnancy-related PRES. Additionally, patients with predisposing risk factors such as intrahepatic cholestasis of pregnancy, known to be associated with a higher risk of preeclampsia, should be closely monitored.

In PRES associated with cytotoxic therapies, endothelial damage leads to capillary leakage and disruption of the blood-brain barrier, ultimately resulting in vasogenic edema. The syndrome can develop even in patients with normal blood pressure and at non-toxic doses. Among the various cytotoxic agents, cyclosporine is one of the most frequently associated with PRES neurological deficits. The same mechanism is observed in PRES associated with chronic renal disease, lupus nephritis and hemolytic uremic syndrome [3].

On the other hand, cerebral autoregulation impairment in acute or severe hypertension leads to cerebral hyperperfusion. This results in the disruption of the blood-brain barrier and leakage of intravascular fluid into the parenchyma, causing cerebral oedema. In this case, both the rate of blood pressure elevation and the level of hypertension are significant factors [9]. Perivascular adrenergic innervation plays a role in autoregulation and is more prominent in the anterior circulation, explaining the preferential involvement of posterior cerebral regions [10]. Although hypertension is common (75% of cases), PRES has also been observed in patients with normal blood pressure [5], such as children who have a more limited range of cerebral autoregulation, and their blood pressure at onset is lower than in adults [11].

The most common clinical symptoms include headaches, altered consciousness, visual disturbances and an epileptic seizure, which is often the first symptom. Only a minority of patients, mostly paucisymptomatic, are seizure-free. Osteotendinous reflexes may be sharp, and Babinski's sign is often present. Some patients can exhibit weakness in the limbs and coordination disorders. Other focal neurological deficits are rare [1,3].

Neuroradiological abnormalities may be visible on non-contrast cerebral Computed Tomography but are best illustrated by MRI angiography. Vasogenic edema in both subcortical and cortical areas is characteristic of PRES and typically appears hypo- or iso-intense on DWI and hyperintense on ADC maps. FLAIR sequences enhance detection sensitivity, highlighting vasogenic edema as a hyperintense signal on the corresponding map. Although the parietal and occipital lobes are the most commonly involved (98%), lesions can be widespread. In a study involving 136 patients conducted by Bartynski and Boardman, other locations were reported, including the frontal lobe with preferential involvement of the superior frontal gyrus (68%, usually with lesions also detected in the posterior lobes). Lesions of the temporal lobes, which are less common, were observed in 40% of patients. The cerebellum (30%) and brainstem (30%) were also affected. Involvement of basal ganglia (13%) and splenium corpus callosum (10%) was described in a minority of patients [12,13].

DWI helps differentiate PRES from early cerebral ischemia. In PRES, vasogenic edema is predominant. However, restricted diffusion, illustrated by hyperintensity on DWI and hypointensity on ADC, indicates cytotoxic edema. This lesion observed in approximately 11% to 26% of cases can be reversible or progress to ischemic injury [3,12], which in our case was limited to the left inferior temporal gyrus. In a series of 36 patients with PRES syndrome and cytotoxic edema reported by Wagih et al., lesions were completely reversible in 32 patients (88.9%), while only four patients (11.1%) progressed to infarction. The transition from cytotoxic edema to infarction is a poorly understood process that remains challenging to predict with imaging [14].

The originality of this case report stems from its wide clinical presentation, marked by persistent memory impairment, a much larger extent of the lesions than the parieto-occipital distribution usually described, with involvement of both temporal lobes along with the presence of cytotoxic edema associated with vasogenic edema. The involvement of both hippocampi (see Figure 2C) explains the amnesia exhibited by our patient, similar to what is observed in hippocampal infarctions. The vascularization of the hippocampi is complex and is supplied by collateral branches of the anterior choroidal artery and the posterior cerebral artery, which is classically involved in PRES syndrome [15]. Moreover, the involvement of the left thalamus, whose vascularization depends on the posterior circulation, also contributes to memory impairment through damage to the mammillothalamic tract (the Vicq d’Azyr circuit, which connects the hippocampus to the thalamus via the mammillary body) [16]. Furthermore, given the extensive cerebral parenchymal involvement, the possibility of an additional diaschisis process cannot be ruled out. These specific lesions are rarely documented in previous studies, highlighting the exceptional nature of this case.

Other medical conditions have been associated with PRES, either due to medications administered for their management or their impact on blood pressure, particularly autoimmune diseases. Sickle cell disease, sepsis, immunomodulatory or immunosuppressive therapies, mainly cyclosporine, are examples of conditions or medications associated with PRES [17].

Most reviews indicate that PRES is generally reversible, with improvement occurring within days to weeks after addressing the underlying cause and managing blood pressure [1,6,12]. Radiological improvement is slower than clinical recovery.

Treatment depends on associated medical conditions and remains symptomatic. In cases of PRES associated with pregnancy, management should also address severe pre-eclampsia. Several studies have shown that PRES associated with eclampsia has a better prognosis compared to other medical conditions [18].

Recurrence of epileptic seizures appears to be rare. In a case series of 127 patients with a recent history of PRES, seizures occurred in eight patients over a median period of 3.2 years. When seizures recur following recovery from PRES, it is appropriate to continue or start antiepileptic treatment [19].

Extensive vasogenic edema, cerebral ischemia, and intracerebral hemorrhage, described in around 20% of patients, are associated with a poor neurological prognosis [20] and may lead to death. In the majority of follow-up investigations, the lesions either diminish significantly or completely heal, pointing towards edema instead of infarction. The recurrence of PRES is rare (<10%) despite the recurrence of contributing factors in these patients [18].

## Conclusions

This case report emphasizes the importance of investigating PRES during the postpartum period when neurological symptoms are prominent despite the absence of hypertension and proteinuria. Early magnetic resonance imaging, especially DWI, is a valuable tool for prompt diagnosis and appropriate management. The long-term prognosis is often favorable, as demonstrated by our patient, who has experienced significant memory recovery after two months, with a maintained quality of life, indicating the likelihood of complete resolution.
